# Evolution and structural diversity of the MotAB stator: insights into the origins of bacterial flagellar motility

**DOI:** 10.1128/mbio.03824-24

**Published:** 2025-09-10

**Authors:** Caroline Puente-Lelievre, Pietro Ridone, Jordan Douglas, Kaustubh Amritkar, Betül Kaçar, Matthew A. B. Baker, Nicholas J. Matzke

**Affiliations:** 1 School of Biological Sciences, University of Auckland 99026, Auckland, New Zealand; 2 Centre for Computational Evolution, University of Auckland 1415, Auckland, New Zealand; 3 School of Biotechnology and Biomolecular Sciences, University of New South Wales 98492, Sydney, Australia; 4 Department of Physics, University of Auckland 533780, Auckland, New Zealand; 5 Department of Bacteriology, University of Wisconsin-Madison 205263, Madison, Wisconsin, USA; University of Cambridge, Cambridge, United Kingdom

**Keywords:** bacterial motility, bacterial flagellum, protein phylogenetics, protein structure, MotAB

## Abstract

The rotation of the bacterial flagellum is powered by the MotAB stator complex, which converts ion flux into torque. Despite its central role in flagellar function, the evolutionary origin and structural diversity of this system remain poorly understood. Here, we present the first comprehensive phylogenetic and structural characterization of MotAB and its closest non-flagellar homologs. We gathered homologs from 205 genomes across 27 bacterial phyla, estimated phylogenies, inferred ancestral sequences, and predicted structures for both extant and inferred ancestral proteins using AlphaFold. Our analyses characterized two structurally distinct groups: flagellar ion transporters (FIT) and generic ion transporters (GIT). FIT proteins are structurally conserved, including a characteristic square fold domain and a torque-generating interface (TGI). We further delineate FIT proteins into two subgroups, TGI4 and TGI5s, based on the presence of 4 or 5 short helices within the TGI region. TGI5 motors, such as those found in the *Escherichia coli* K12 system, are primarily restricted to Pseudomonadota, whereas TGI4 motors, such as the Na^+^-powered polar motors of *Vibrio* (PomAB), are distributed across a broader range of bacterial lineages. In contrast, GIT proteins exhibit substantial structural and functional heterogeneity and lack features associated with flagellar motility. Nevertheless, a conserved interaction between the A and B subunits is retained across FIT and GIT proteins, with their corresponding genes typically adjacent to operons. Functional assays in *E. coli* show that FIT-specific structural elements are indispensable for flagellar motility. Our results suggest that the flagellar stator motor complex evolved once from a common ancestral ion transporter, acquiring unique structural traits to support motility. This work provides a robust framework for understanding the evolutionary diversification of stator complexes and their mechanistic specialization.

**IMPORTANCE:**

Flagellar motility allows bacteria to propel themselves and direct movement according to environmental conditions. It plays a key role in bacterial pathogenicity and survival. We investigated the molecular and structural diversity of the stator motor proteins that provide the ion motive force to power flagellar rotation. This study uses a comparative approach that integrates phylogenetics, 3D protein structure, motility assays, and ancestral state reconstruction (ASR) to provide insights into the structural mechanisms that first powered the flagellar motor. We provide the first phylogenetic and structural characterization and classification of MotAB and relatives.

## INTRODUCTION

Bacterial flagellar motility is one of the most ancient and widespread forms of cellular movement (1). It allows bacteria to propel themselves and direct their movement by rotating their flagella (2). Flagellar rotation is driven by the bacterial flagellar motor (BFM), a membrane-embedded rotary nanomachine that harnesses the ion motive force (IMF) to generate torque (3). Central to this process is the stator complex, a multimeric assembly that couples transmembrane ion flux to motor rotation (4).

Flagellar stators are usually composed of five A subunits (MotA or PomA) and two B subunits (MotB or PomB), forming a proton or sodium-driven ion channel, respectively (5–7). The ion binding channel is made of two transmembrane regions in the A subunit (A-TM3 and A-TM4) and the transmembrane region of the B subunit, whereas the A-TM2 helix serves as a membrane anchor for the cytoplasmic torque-generating interface. To couple ion flow to motor rotation, the stator directly interacts with the rotor protein FliG at conserved residues (R90/D288-D289 and E98/R281 in *Escherichia coli*) (4, 8).

Ion conduction through the flagellar stator system is regulated by the plug domain, a short periplasmic region adjacent to the B subunit’s transmembrane helix. The plug is essential for motor activation and acts as a gate to prevent premature ion leakage until the stator is correctly docked to the motor (6, 9, 10).

Despite their central role in bacterial motility, the evolutionary origins of flagellar stators remain poorly resolved. Their broad distribution across gram-positive and gram-negative bacteria suggests an early origin, possibly dating back to the last universal common ancestor (11). Flagellar stators share homology with non-flagellar IMF-powered systems such as ExbBD (12–14), which energizes TonB-dependent uptake of iron and other nutrients at the outer membrane (15) and TolQR of the Tol-Pal system, which functions in cell division and outer membrane homeostasis (16). Previous phylogenetic analyses showed that ExbBD and TolQR form distinct clades (11) but did not analyze numerous other homologs that appear in databases. A scenario in which the last common ancestor of Bacteria was a gram-negative diderm would support an evolutionary pathway from ExbBD/TolQR-like transporters to torque-generating stators via exaptation (2, 17).

A systematic analysis of structural features that differentiate flagellar stators from their non-flagellar counterparts has not yet been conducted within a phylogenetic framework. Structural innovations such as disrupted transmembrane helices, expanded torque-generating interfaces, specialized C-terminal domains, and variability in the plug domain could be particularly significant. Identifying how these traits emerged may clarify the evolutionary transition from generalized ion transporters to specialized torque-generating complexes. Such insights could bring to light broader patterns of molecular innovation and evolutionary adaptation, revealing how bacterial proteins diversify structurally and functionally to exploit new ecological niches and survival strategies.

The aim of this study was to determine the phylogenetic relationships of the MotAB stator complex and its non-flagellar homologs and to use this evolutionary framework to describe the structural diversity of bacterial proton-coupled transmembrane transport systems. We integrated deep homology searches, structure-informed sequence alignments, and gene neighborhood analyses with Bayesian phylogenetic inference and ancestral sequence reconstruction. AlphaFold structural predictions for extant and ancestral proteins were used to trace the emergence of key molecular features. Finally, we tested the functional significance of a major structural innovation, the MotA Torque Generating Interface, through targeted deletions and motility assays in *Escherichia coli*. Together, our results shed light on the evolutionary trajectory of the stator complex and the structural changes that enabled the transition to flagellar motility.

## RESULTS

### The A and B subunits occur in operons across diverse bacterial genomes

**Fig 1:**
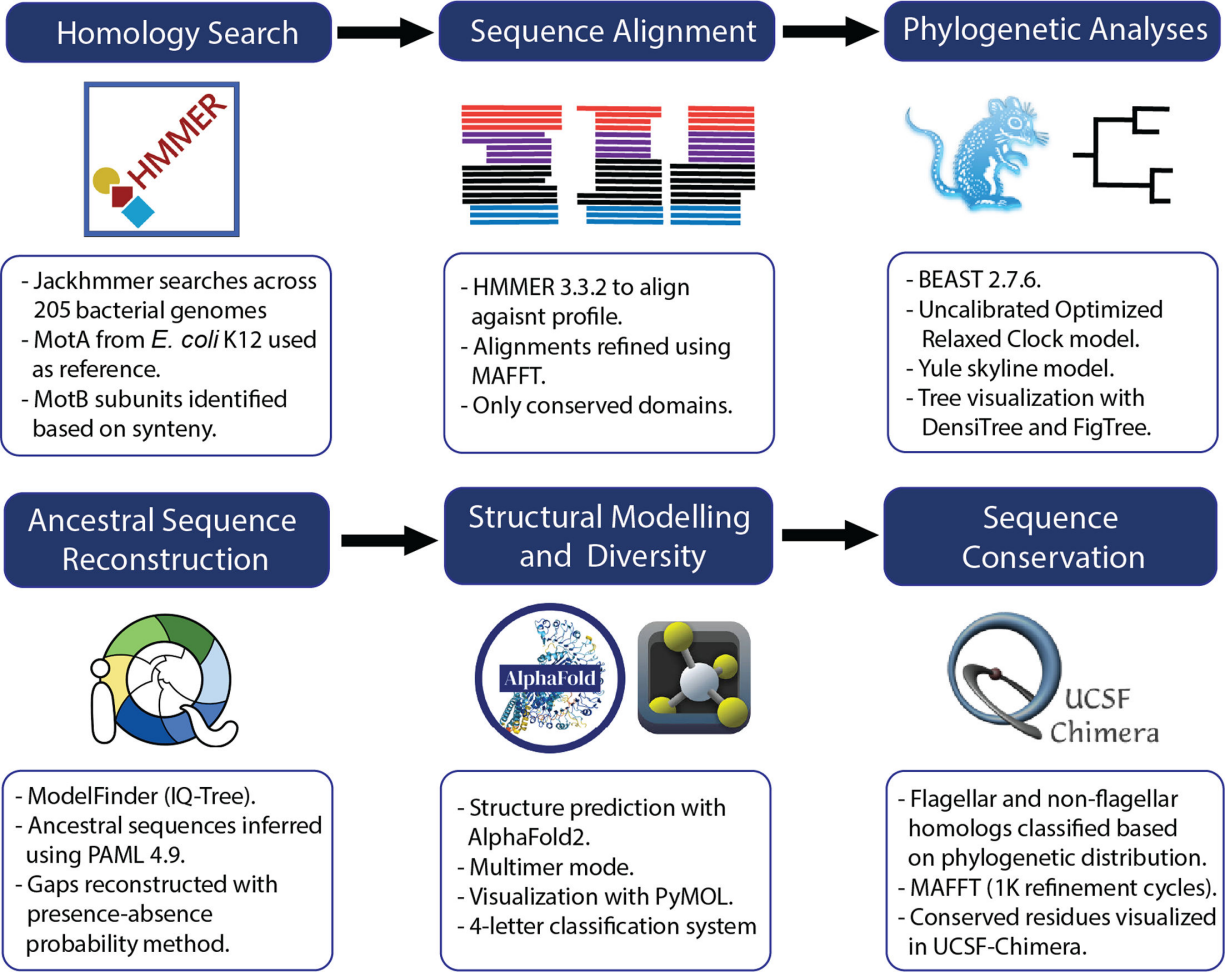
Workflow diagram summarizing the methodological pipeline used in this study. The pipeline includes homology searches using Jackhmmer, sequence alignment with HMMER and MAFFT, phylogenetic inference using BEAST with relaxed clock models, ancestral sequence reconstruction via IQ-TREE and PAML, structural classification using experimental structures and AlphaFold2, and sequence conservation analysis visualized with UCSF-Chimera. Each step outlines the specific bioinformatics tools and analytical procedures implemented.

We conducted homology searches using Jackhmmer across 205 sampled bacterial genomes (Fig. 1), identifying 746 candidate homologs for MotA. After manual curation to exclude sequences below 10% similarity and false positives, we retained 379 sequences for further analysis. Homology searches for the corresponding B subunits, either based on their full-length sequences or their conserved transmembrane (TM) domains, proved challenging due to their lower sequence conservation. Proteins annotated as MotB or OmpA-like were readily identified, whereas known structural homologs, such as ExbD and TolR (12), typically fell below detection thresholds. Consequently, we inferred the B subunit identities based on genomic proximity to MotA homologs. In most analyzed genomes (excluding those cases where the A subunit was duplicated), the B gene consistently appeared directly downstream of the A gene, indicating strong conservation of operon structure. Alignment produced a final data set with 295 MotA columns and 308 MotB columns. MotA sequences displayed a higher mean pairwise identity (17.7%) compared with MotB (10.6%).

### Flagellar stators form a single evolutionary group distinct from non-flagellar homologs

We evaluated congruence between phylogenies inferred from the separate A and B subunit data sets using clade support entropy, a metric quantifying uncertainty across phylogenetic trees (18). BEAST2 analyses (Fig. 1) identified greater uncertainty in the B subunit phylogeny, with 71,518 recovered clades and high entropy (256.7 natural log units), compared with the A subunit’s 3,449 clades and lower entropy (105.8). DensiTree visualizations show that deep branches within the B subunit trees lacked robust statistical support. By concatenating the A and B subunit data sets, we reduced uncertainty, recovering 1,998 clades with significantly lower entropy (90.8) and increased posterior support for deep relationships (Fig. S1).

**Fig 2:**
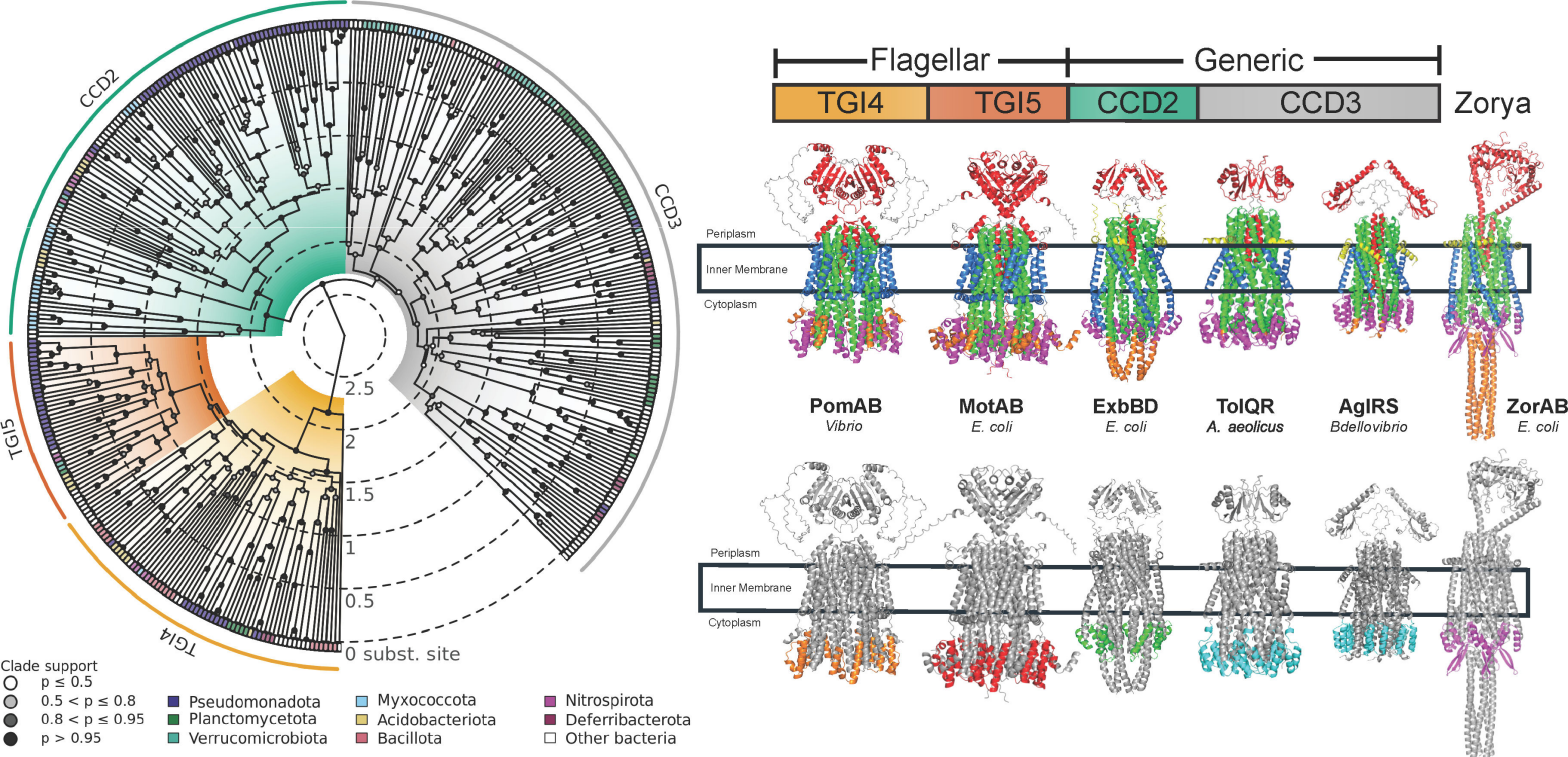
Phylogenetic relationships of the bacterial flagellar stators and their non-flagellar homologs. Concatenated Bayesian phylogeny of the A and B subunits. Branch lengths are scaled in substitutions per site (inner concentric rings mark 0.5 unit intervals). Circles on nodes represent posterior probability (white: *P* ≤ 0.5; gray: 0.5 < *P* ≤ 0.8; dark gray: 0.8 < *P* ≤ 0.95; black: *P* > 0.95). The outer ring is color-coded to represent bacterial phyla (legend at bottom left). Major clades are indicated by the colored arcs outside the tree: TGI4 (gold): flagellar stator complexes with a four-helix torque-generating interface (e.g., PomAB); TGI5 (orange): flagellar stator complexes with a five-helix Torque-generating interface (e.g., MotAB); CCD2 (green): non-flagellar generic ion transporters with a two-helix condensed cytoplasmic domain; CCD3 (light gray): non-flagellar generic ion transporters with a three-helix condensed cytoplasmic domain. Multimeric structures for representative AB complexes are arranged left to right to match the colored clades in the phylogeny. The upper row shows the full transmembrane assembly with both A and B subunits colored as follows: N-terminus in yellow, transmembrane helices in blue/green, cytoplasmic domains in magenta, C-terminus in orange, and B periplasmic domains in red. The lower row shows each complex as a transparent gray outline with only the cytoplasmic domains highlighted to emphasize structural differences. From left to right: PomAB (*Vibrio*): TGI4 proton/sodium-driven stator, MotAB (*E. coli*): TGI5 proton-driven stator, ExbBD (*E. coli*): CCD2 TonB system homolog, TolQR (*E. coli*): CCD3 Tol-Pal system homolog, AglRS (*Bdellovibrio*): CCD3 gliding-motility homolog, ZorAB (*E. coli*): remote phage-defense-related homolog (shown for structural comparison but excluded from phylogenetic analyses).

The resulting phylogeny clearly delineated two major groups (Fig. 2). The first clade comprises flagellar-associated MotAB complexes such as the well-characterized *E. coli* MotAB and *Vibrio* PomAB systems. We term this clade the bacterial flagellar ion transporters (FIT). The second clade, termed bacterial generic ion transporters (GIT), includes well-studied structural homologs such as ExbBD and TolQR from *E. coli*, along with proteins associated with non-flagellar functions, such as gliding motility proteins AglRS (19), and other proteins with unknown function. Among the 379 proteins analyzed, 107 belonged to FIT and 272 to GIT. The FIT clade subdivided into two structurally distinct groups: (i) TGI4, found across both gram-positive and gram-negative, H+ and Na+ powered bacteria (e.g., *Vibrio* PomAB), including Aquificae, Pseudomonadota, Bacillota, Spirochaetes, Planctomycetota, Acidobacteria, Deferribacteres, Chloroflexi, and Nitrospirae, and (ii) TGI5, a derived group within TGI4, predominantly found in gram-negative, H+ powered Pseudomonadota (e.g., *E. coli* MotAB) and other phyla (Planctomycetota, Verrucomicrobia, Armatimonadetes, Gemmatimonadetes, Acidobacteria, and Nitrospirae) (Fig. 2).

### Flagellar-specific structural traits are conserved across diverse bacteria

**Fig 3:**
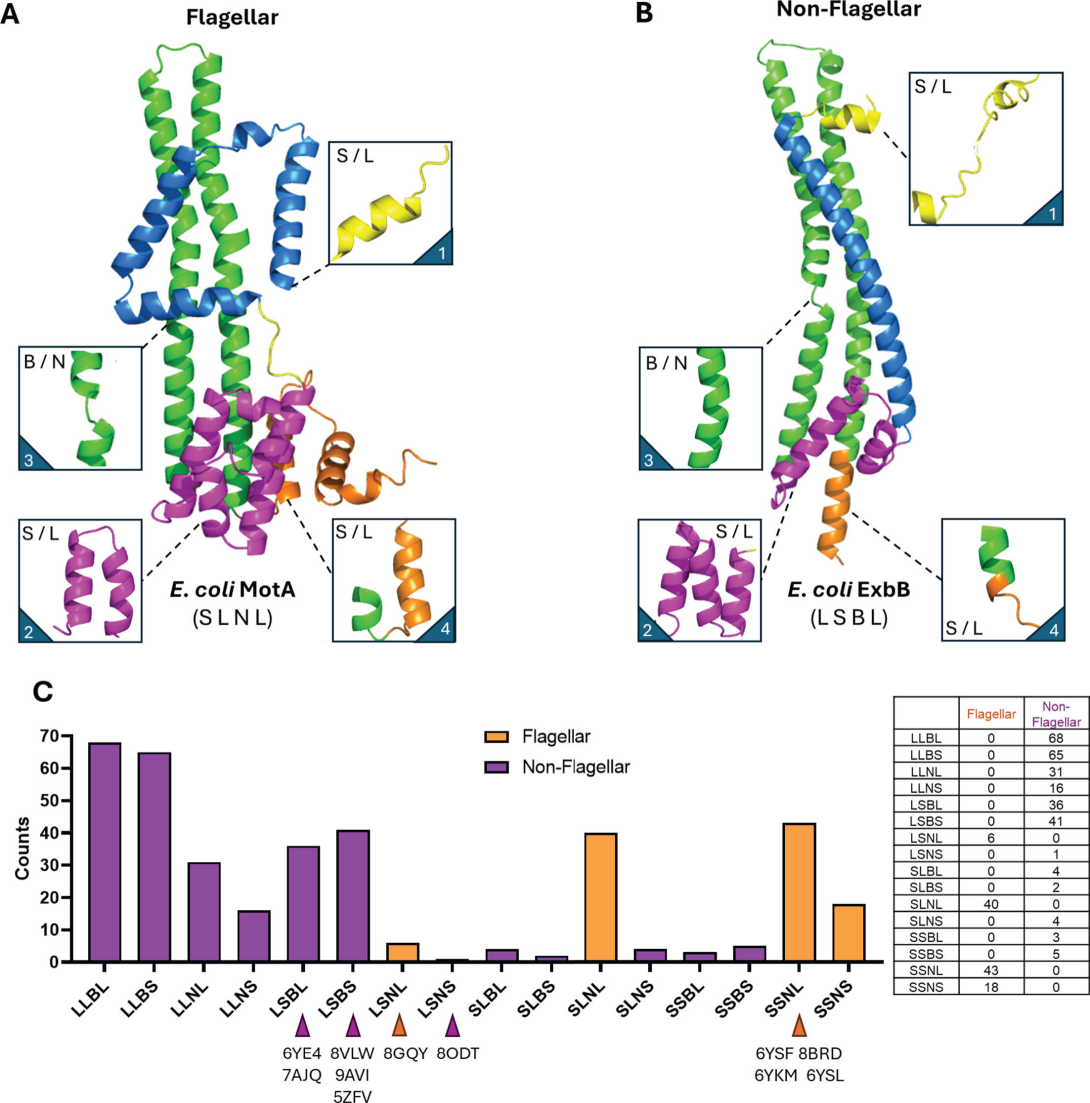
Structural classification of A-subunit homologs. Monomeric models of (**A**) *E. coli* MotA and (**B**) *E. coli* ExbB colored based on structural features such as the N-terminal domain (yellow), TM domain (blue), the TGI domain (pink), TM3/4 (green), and C-terminal domain (orange). For each homolog, four regions of the protein were considered for classification purposes: an N-terminal appendage (1, small or large), the TGI domain (2, small or large), TM3 (3, broken or not broken), and the C-terminal domain (4, small or large). Each homolog was expressed as a four-letter code, as exemplified below each model (MotA: SLNL, ExbB: LSBL). (**C**) Bar chart summary of the classification for the whole A-subunit phylogeny. Entries belonging to the non-flagellar (purple) and flagellar (orange) clades were categorized and counted according to the four-letter code shown on the *x*-axis.

**Fig 4:**
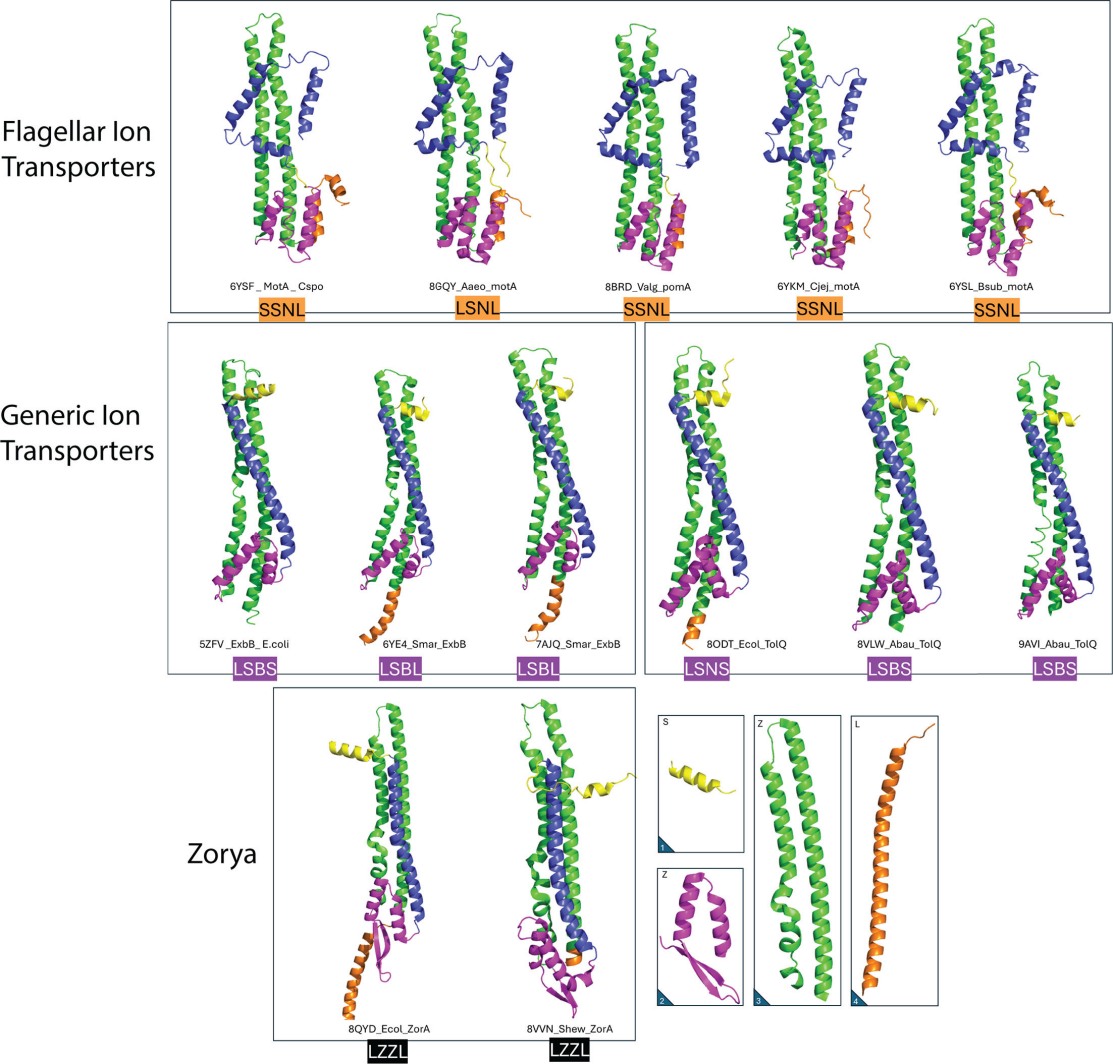
Structural diversity of MotA homologs based on experimentally determined protein structures representing the variations underlying the functional diversification of bacterial stator complexes. Each structure was categorized using a four-letter code reflecting four key structural characteristics: 1, size of the N-terminal appendage (S: small, L: large; yellow); 2, size and complexity of the torque-generating interface (S: ≤2 helices, Z: two helices and a beta-sheet unique to Zorya-type proteins, L: ≥3 helices; magenta); 3, integrity of transmembrane helix 3 (N: intact, B: broken; green); and 4, size of the C-terminal domain (S: small, L: large; orange). Structural elements are colored as follows: N-terminal appendage (yellow), transmembrane helices (blue and green), torque-generating interface (magenta), and C-terminal domain (orange). Orange labels indicate flagellar, purple indicates non-flagellar, and black indicates Zorya proteins. The Zorya-type proteins are distinguished by the presence of additional beta-sheet structures in their cytoplasmic domains. Insets highlight individual domains characteristic of the Zorya proteins.

**Fig 5:**
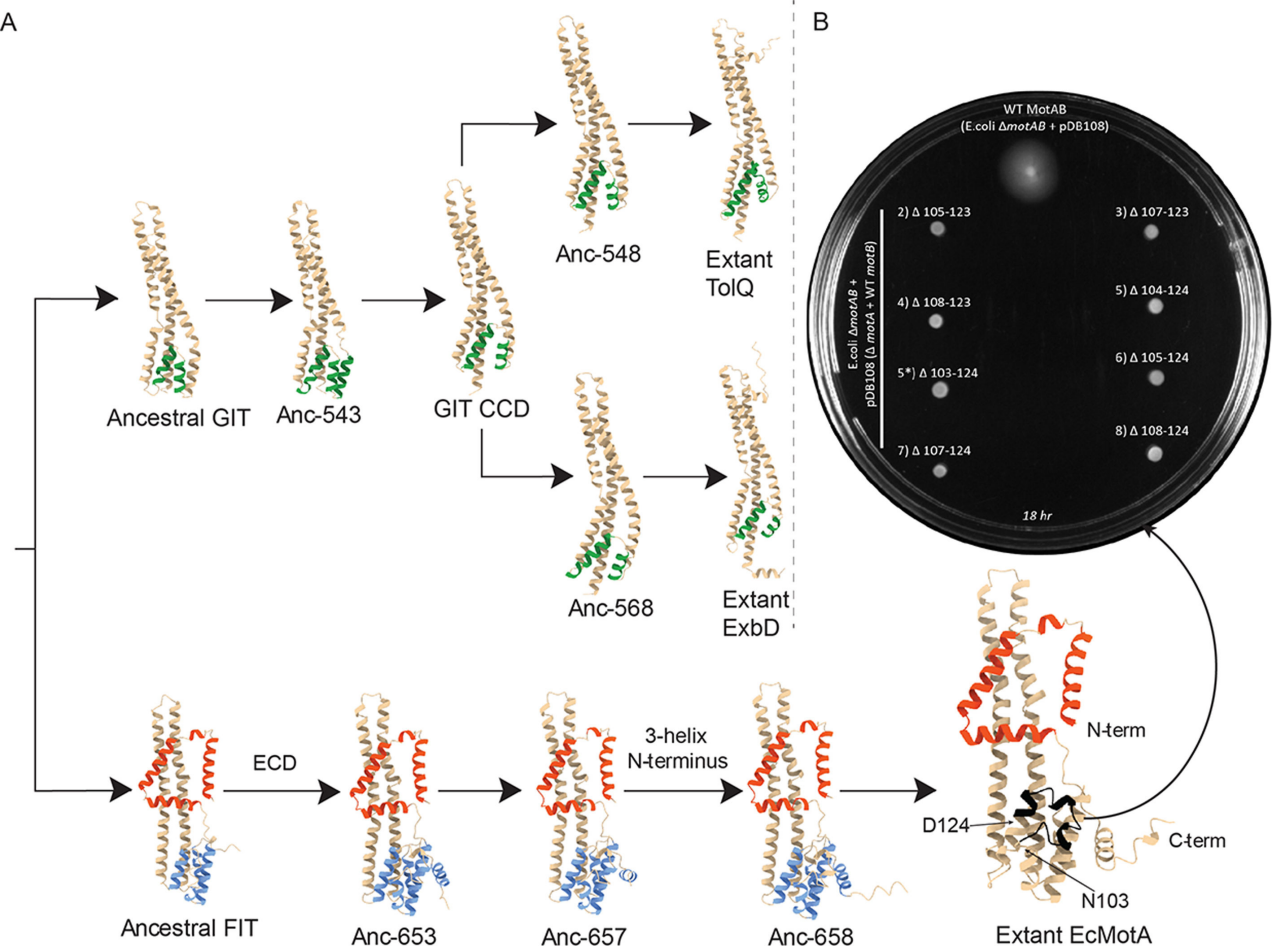
Evolutionary trajectory and functional validation of structural features in the bacterial FIT and GIT. (**A**) Predicted structural models of ancestral proteins (nodes 381, 543, 547, 548, 568, 652, 653, 657, and 658 from Fig. S4) illustrate the emergence of structural innovations specific to flagellar motility (FIT lineage) and the structural diversity observed in non-flagellar homologs (GIT lineage). (**B**) Soft-agar motility assay shows that partial deletions of the torque-generating interface (TGI) domain (residues N103–D124, highlighted in black in the AF2 model of *E. coli* MotA) abolish motility in an *E. coli* ΔmotAB strain. Plates inoculated with wild-type (WT) MotAB show motility, whereas all TGI-deletion mutants (variants 2–9) fail to form swim rings after incubation for 18 hours at 30°C on LB agar plates (0.25%, 85 mM Na^+^).

**Fig 6:**
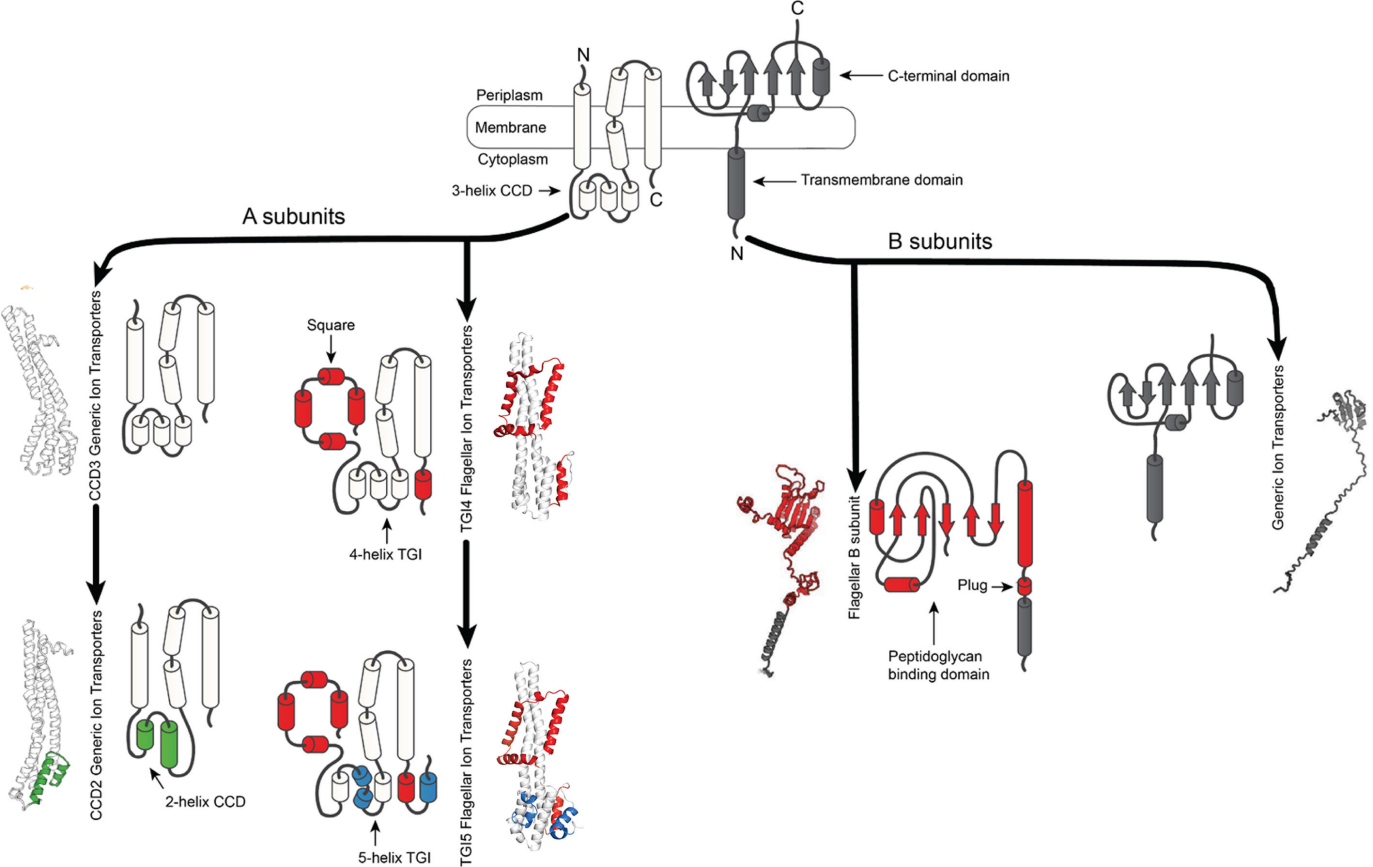
Simplified evolutionary model illustrating structural changes in the A and B subunits of the bacterial ion transporters and their specialization in flagellar motility. Highlighted are the key structural innovations: torque-generating interface (TGI), the square fold domain, the plug + linker domain, and the OmpA-related expanded peptidoglycan binding domain (EPGB), which differentiates the FIT from its GIT relatives. These features likely evolved and became functionally specialized to support bacterial flagellar rotation.

To systematically investigate structural features, we generated monomer AlphaFold models for all 379 A and B subunits. Comparative structural analyses revealed specific conserved features unique to FIT proteins and absent from GIT homologs (Fig. 3 to 6). These include (i) an expanded cytoplasmic domain (ECD), notably, a torque-generating interface (TGI) comprising at least four helices in the A subunit (Fig. 3), (ii) a distinctive square-shaped four-helix transmembrane domain (square fold) in the A subunit (Fig. 4), (iii) a plug + linker domain adjacent to the TM helix in the B subunit (Fig. 2), and (iv) an expanded peptidoglycan binding (EPGB) domain (homologous to OmpA) in the periplasmic region of the B subunit (Fig. 6). Within the FIT group, structural divergence between TGI4 and TGI5 subclades primarily involved the TGI helices count. TGI5 stators exclusively featured MotB-like plugs, whereas TGI4 stators included both MotB- and PomB-like plugs. Sequence conservation was notably lower in PomB-like plugs (Fig. S2). Overall, FIT proteins showed limited structural variation, with only five out of 16 theoretically possible combinations of these structural elements observed (Fig. 3). Sequence conservation analysis further highlighted strong residue conservation in the TM domains (Fig. S3), consistent with their critical roles in subunit interactions and ion channel formation (4).

### Non-flagellar GIT complexes show structural diversity and variability

In contrast to the conserved FIT architecture, GIT proteins exhibited remarkable structural heterogeneity. GIT complexes typically lacked the plug + linker domain in their B subunit, and their A subunits frequently presented diverse extensions at the N- and C-terminal regions (Fig. 3 and 4). Additionally, their cytoplasmic domains were notably condensed, with either two (CCD2, e.g., ExbB) or three (CCD3) helices (e.g., TolQ and AglR) (Fig. 2). This diversity resulted in 13 distinct structural combinations, from a total of 16 theoretically possible variations, and included proteins with extraordinarily large periplasmic domains (Fig. 3).

### Ancestral reconstruction reveals early structural divergence

AlphaFold predictions of ancestral protein sequences revealed clear divergent paths for FIT and GIT complexes. The ancestral FIT protein had a square-fold TM domain and a small (3-helix) cytoplasmic domain. Subsequently, progressive expansion of the cytoplasmic domain to form distinct TGI4 and TGI5 subclades evolved within FIT. In contrast, ancestral GIT proteins lacked these structural signatures, exhibiting linear TM domains and significantly reduced CCD elements, suggesting an early and fundamental divergence (Fig. 5).

A critical structural difference highlighted by our analysis was the presence of breaks in the TM helices of non-flagellar proteins, specifically in the TM3 region (Fig. 3; Fig. S3). Such breaks were consistently absent in FIT proteins, which maintained a highly conserved TM helix structure. Sequence and structural comparisons in Fig. S3 illustrate these distinctions.

**Fig 7:**
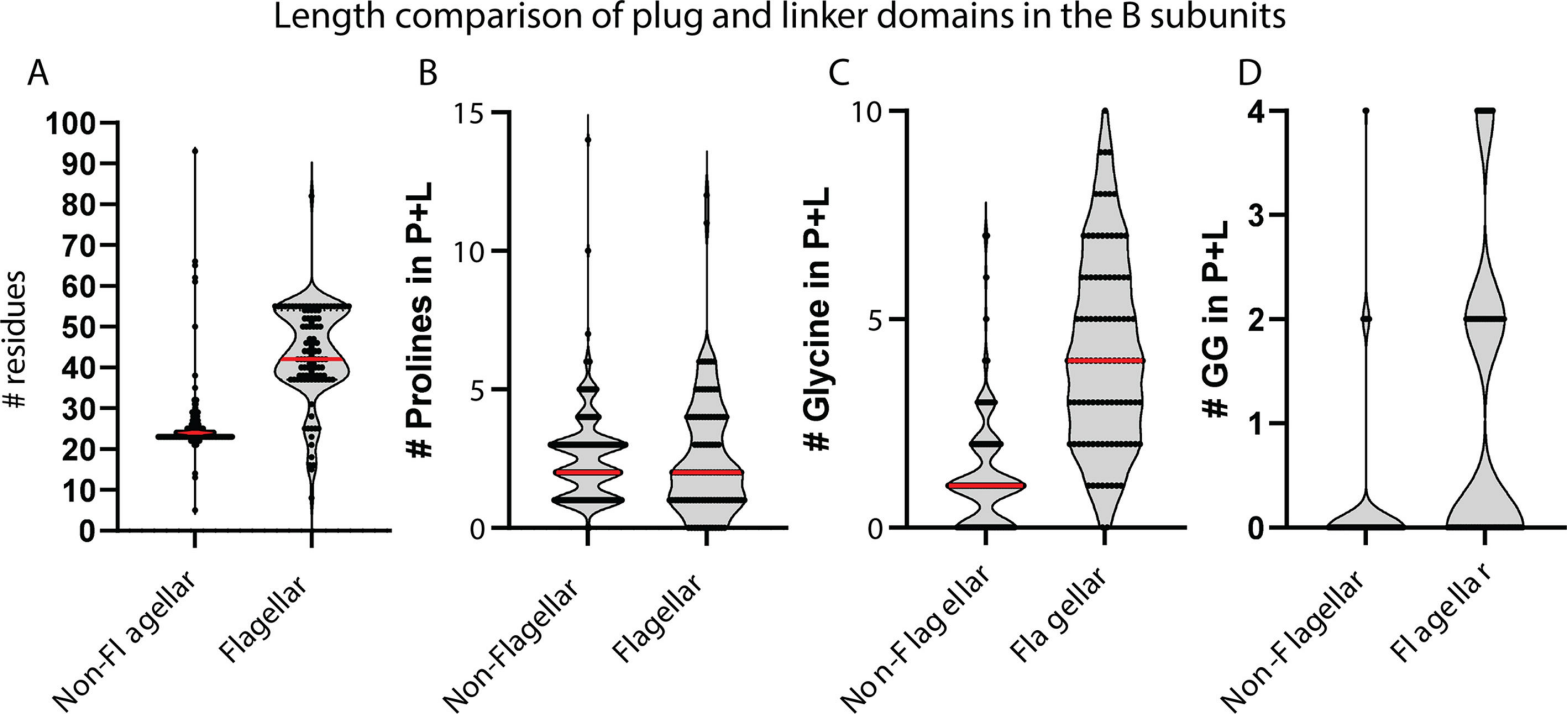
Comparative analysis of plug + linker domain length and glycine composition in flagellar and non-flagellar B subunits. (**A**) Violin plots showing the distribution of plug + linker domain lengths across six stator groups. Flagellar ion transporters (FIT: TGI4 and TGI5) exhibit significantly longer plug + linker regions compared with generic ion transporters (GIT: CCD2, CCD3), suggesting adaptations for increased conformational flexibility. (**B**) Distribution of proline residue counts within the plug + linker domain. (**C**) Distribution of glycine residue counts within the plug + linker domain. (**D**) Distribution of GG counts within the plug + linker domain. FIT groups show a significantly higher proline and glycine content than GIT groups, consistent with enhanced gating flexibility. Red bars indicate median values; black dots represent individual protein sequences.

Furthermore, the plug domain of the B subunit showed clear differences between FIT and GIT proteins. FIT proteins consistently exhibited defined plug + linker domains with longer sequence length and were typically enriched with glycine residues. Conversely, GIT proteins showed significantly greater variability in sequence length and generally reduced glycine content, correlating with reduced structural flexibility (Fig. 7).

### The TGI5 domain is essential for flagellar motility in *Escherichia coli*

To experimentally validate the functional significance of the TGI domain, we created eight *E. coli* MotA variants with partial deletions spanning residues N103–D124, a region of the protein encompassing TGI5-specific structural features. Each variant was expressed alongside wild-type MotB in a motility-deficient *E. coli* ΔmotAB strain and tested for motility rescue using soft-agar swim assays. None of the partially deleted MotA variants restored motility, whereas control strains expressing wild-type MotAB produced typical swim rings (Fig. 5; Fig. S4). These results demonstrate that the TGI5 region is essential for flagellar-driven motility in this strain.

## DISCUSSION

In this study, we provided a phylogenetic and structural characterization of the MotAB stator complex and its homologs across a broad phylogenetic sampling of bacterial lineages. Using an integrative approach combining homology searches, Bayesian phylogenetics, ancestral sequence reconstruction, AlphaFold structural predictions, and experimental validation, we identified critical structural traits that distinguish flagellar ion transporters (FIT) from their generic homologs (GIT). We found strong evidence supporting a single evolutionary origin for flagellar stators, characterized by conserved structural innovations essential for their specialized function in motility.

### Structural innovations driving flagellar stator specialization

The conserved square-fold transmembrane (TM) architecture and the ECD, specifically the TGI, emerged as diagnostic traits for FIT proteins. Notably, FIT proteins exhibit a remarkably high degree of residue conservation in their TM domains, and unlike their non-flagellar GIT homologs, consistently maintain unbroken transmembrane helices (Fig. S3). Such structural integrity could contribute to their specialized function in tightly controlled ion conduction and efficient torque generation. Experimental assays confirmed that deletion of the TGI5-specific residues in *E. coli* MotA completely abolishes motility, underscoring its critical functional role (Fig. 5). Structural divergence within FIT proteins, particularly between TGI4 and TGI5 subgroups (Fig. 6), further suggests potential adaptations in rotor-stator interactions and regulatory mechanisms, likely reflecting niche-specific selective pressures or adaptations related to ion usage (8, 20, 21). Comparative studies of TGI4 and TGI5 rotor-stator complexes will clarify how structural variation translates into distinct functional outcomes and regulatory strategies across diverse bacterial taxa.

### From TGI4 to TGI5: structural divergence within flagellar stators

**Fig 8:**
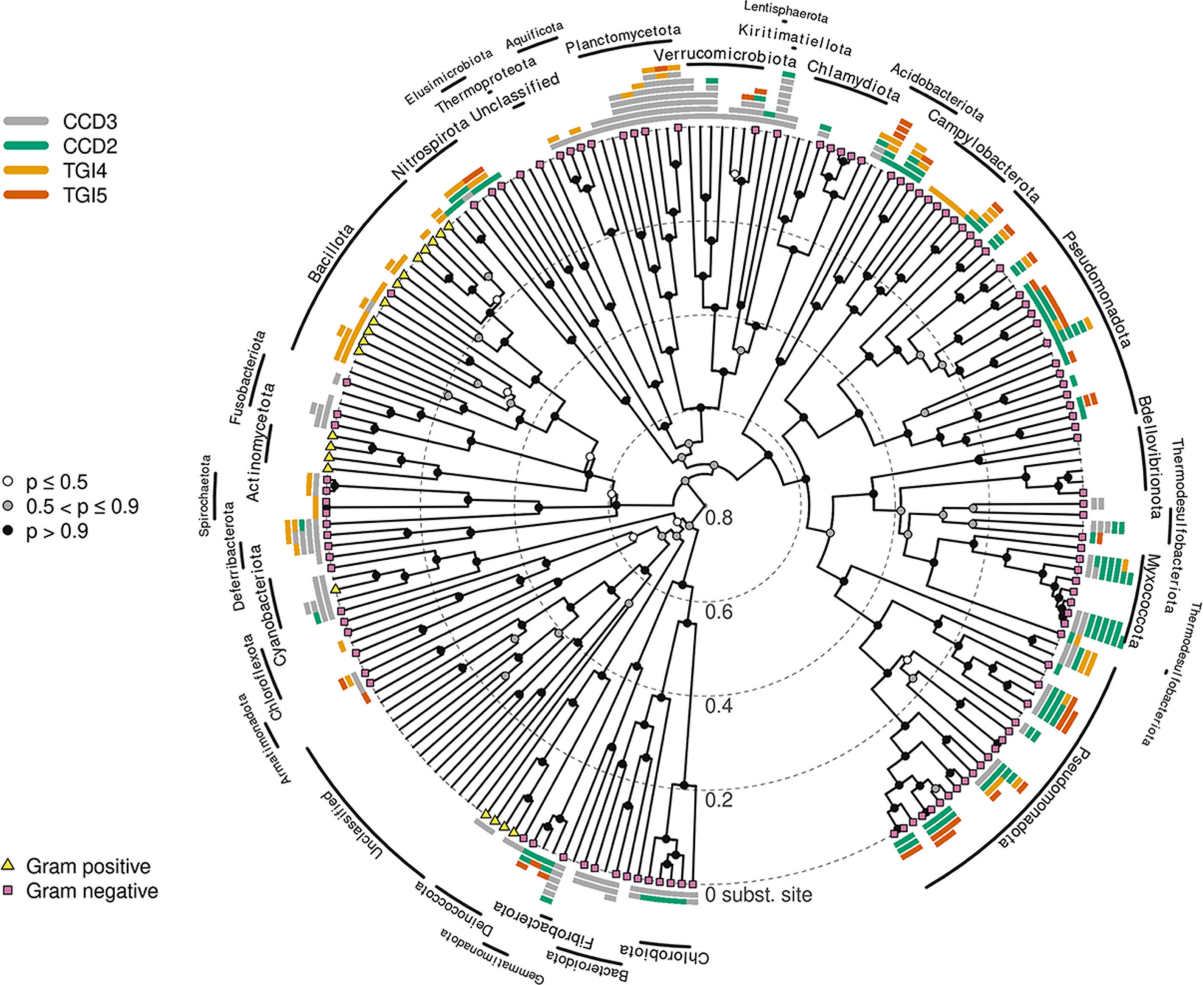
Species (16S rRNA) phylogeny showing distribution and structural classification of MotAB homologs across bacterial phyla. Distribution of structural motifs is highlighted as follows: CCD2 (two-helix condensed cytoplasmic domain), CCD3 (three-helix condensed cytoplasmic domain), TGI4 (four-helix torque-generating interface), and TGI5 (five-helix torque-generating interface). Gram-positive (yellow triangles) and gram-negative (purple squares) bacterial taxa are indicated at the tips, according to reference 22. Node circles represent posterior probability values (*P*): white (≤0.5), gray (0.5 < *P* ≤ 0.9), and black (>0.9). Bacterial phyla are labeled around the perimeter.

Phylogenetic and structural analyses suggest that the TGI5 stators are derived from a TGI4-type stator (Fig. 2), representing a significant structural innovation within the FIT lineage. The ancestral FIT proteins likely had TGI4-like structural characteristics such as a torque-generating helix (Fig. 6). The emergence of the TGI5 domain involved an expansion of the torque-generating interface with an additional helix, potentially enhancing rotor-stator interactions and torque generation efficiency. This structural modification is present only in gram-negative taxa, primarily Pseudomonadota, including proton-driven systems such as the *E. coli* MotAB system (Fig. 2 and 8), suggesting a potential adaptive shift toward higher torque generation or more refined motor regulation. Future experimental studies comparing rotor-stator dynamics between TGI4 and TGI5 systems could further clarify the functional significance of this evolutionary transition and its ecological implications (8, 21).

### Functional and evolutionary diversity of generic ion transporters

Unlike the highly conserved FIT stators, GIT proteins display remarkable structural plasticity (Fig. 3, 4, and 7). Their A subunits can include additional N-terminal and C-terminal domains, many of them of unknown function (e.g., DUF3450 and DUF2341). We hypothesize these “extra” domains have been co-opted for lineage-specific functions rather than rotor interactions. Moreover, GIT B subunits lack the extended plug + linker and EPGB motifs (homologous to OmpA) present in FIT B subunits (Fig. 6). They carry instead a more compact peptidoglycan-binding fold (we could discern no homology to OmpA) that is unlikely to support high-torque anchoring.

The assortment of DUFs (domain of unknown function) appendages suggests repeated, independent acquisitions onto a shared Exb/Tol scaffold, driving functional novelties beyond motility, such as nutrient uptake, envelope stress responses, or phage defense, as in the even more distantly related Zorya systems (23). The stark differences in plug + linker/C-terminal architecture (Fig. 7; Fig. S2) between GIT and FIT further imply that the FIT plug + linker domain was a key innovation for torque regulation, whereas GIT retained a minimal periplasmic module optimized for diverse, low-torque tasks.

### Evolutionary significance of the plug domain

Our results highlight the evolutionary and functional importance of the plug domain in the flagellar B subunits. Functionally, the plug regulates ion flux by acting as a gate that physically blocks the ion channel in the absence of motor engagement, thereby preventing uncontrolled ion leakage across the inner membrane (5, 6). Upon docking with the rotor complex, conformational changes in the stator reposition the plug, opening the ion-conducting pathway and enabling torque generation (24).

FIT proteins consistently have plug + linker domains that are both longer and significantly enriched in glycine residues compared with their GIT homologs (Fig. 7). This observation is supported by a comparative analysis across stator clades (Fig. S2), where both length and glycine content were significantly higher in FIT subunits (*P* < 0.05). The enrichment of glycine, a residue associated with backbone flexibility (25), suggests that the FIT plug + linker region is related to dynamic conformational changes required for fast and reversible gating during motor activation.

On the other hand, GIT proteins typically lack a clearly defined plug domain and instead exhibit shorter and more constrained periplasmic regions (Fig. 2 and 6). This structural difference further supports the hypothesis that the plug domain is a specialized trait associated with flagellar motility, enabling precise and conditional control of ion flow in response to rotor engagement. The presence and conservation of these features across the FIT lineage highlight its evolutionary importance in the emergence and specialization of flagellar motility.

### Continuous transmembrane domains (TM3) in flagellar stators vs kinked in non-flagellar homologs

Another important structural difference between the FIT and GIT stators is in the integrity of their transmembrane helices (Fig. 3 and 4). In FIT proteins, TM3 remains a continuous helix, whereas in GIT homologs (e.g., ExbB, TolQ), TM3 is interrupted by a pronounced kink or “break” (Fig. 3). This break varies in length across GIT (Fig. 4). The local sequence alignment of the residues flanking the TM3 break shows a conserved Glu–Pro (E-P) triad motif in FIT that directly faces the fourth helix of the square‐fold transmembrane domain, with the proline side chain oriented toward helix 4 of MotA (Fig. S2). This arrangement potentially stabilizes inter-subunit interactions and conformational changes during torque generation. In contrast, GIT proteins such as ExbB and TolQ display disrupted TM3 helices exposed to solvent and enriched in positively charged residues (Arg and Lys) at equivalent positions (*Ec* ExbB R128). No stabilizing intramembrane helices are found in front of the break, and the flanking region around the kink is more disordered (Fig. S2).

These differences suggest that TM3 in MotA plays a role in regulating intramembrane conformational shifts, potentially associated with the torque generation. Conversely, the TM3 break in GIT proteins may act as a flexible hinge involved in broader conformational transitions linked to, for example, energizing other components (e.g., TonB or TolA) rather than rotation. These differences highlight how structurally homologous domains could evolve distinct mechanisms for coupling ion flow to function, with FIT stators favoring mechanical rigidity for torque transmission, and GIT complexes favoring flexibility for energy transduction.

### Conclusions

Our findings provide the most robust phylogenetic and structural framework to date to further understand the evolutionary history and functional specialization of bacterial ion transporters. Future experimental studies could leverage our proposed classification system to investigate uncharacterized homologs and clarify their ecological roles. Additionally, exploring the function of uncharacterized domains identified in GIT proteins, particularly N-terminal appendages (e.g., DUF3450 and DUF2341), could uncover novel biological functions and regulatory mechanisms. Finally, integrating structural characters directly into phylogenetic inference methods (26) promises even deeper insights into ancient protein evolution, potentially resolving the evolutionary placement of more remote homologs such as the recently characterized ZorAB (23) and GldLM complexes (27).

**Fig 9:**
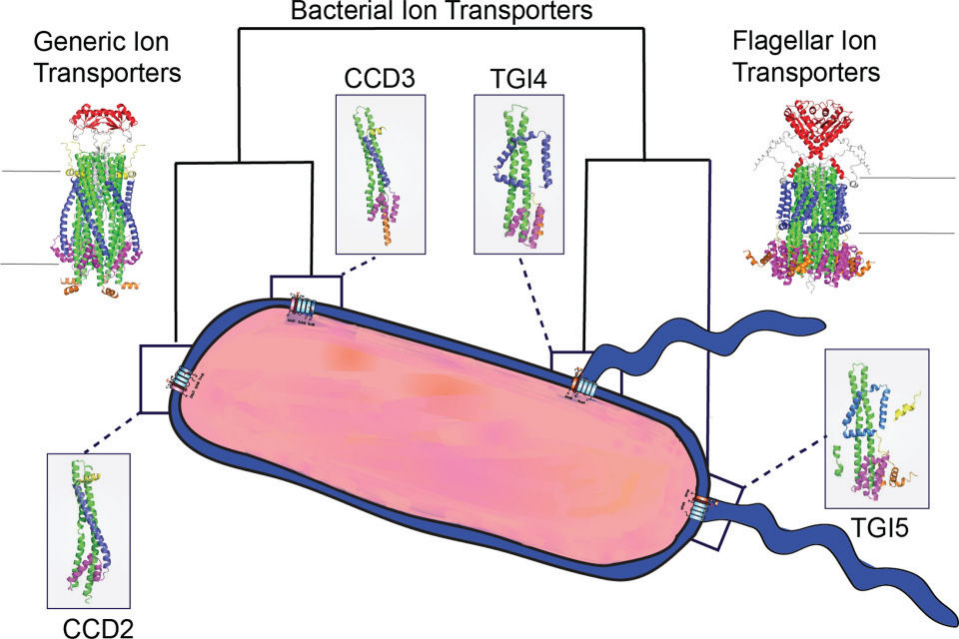
Overview of rotary bacterial ion transporters. Motility-associated FIT (TGI4, TGI5) form a specialized lineage within a broader family of bacterial ion transporters. These flagellar stators share common ancestry with GIT (CCD2, CCD3) and have evolved structural features that enable the transfer of torque to drive flagellum rotation.

In summary, our integrative study identifies key structural innovations that facilitated the evolutionary transition from generic ion transport systems to highly specialized torque-generating stators, enhancing our understanding of how molecular specialization arises in bacterial systems (Fig. 9). This approach highlights the power of combining structural, phylogenetic, and functional analyses to elucidate deep evolutionary patterns and mechanisms in molecular biology.

## MATERIALS AND METHODS

### Homology search and phylogenetic analyses

A total of 205 fully annotated and complete bacterial genomes were selected to maximize taxonomic representation, including both motile and non-motile taxa from 27 phyla. When genome annotation was not available from the NCBI (28), genomes were annotated using Prokka (29). The MotA sequence data set was assembled using HMMER 3.3.2 (November 2020; http://hmmer.org/) to perform homology searches with MotA from *E. coli* K12 as a reference (GenBank accession no. AAC74960.1). Jackhmmer was run for five iterations with the default search parameters. Since the genes for the A and B subunits are typically in synteny and function in an operon, the B subunits were identified with the gene immediately downstream from A. Identification of B subunits was confirmed manually with GenBank annotations and alignments. Protein sequences were initially aligned against a tailored HMMER profile that was generated from these data sets. Alignments were then refined with MAFFT (30, 31). Final alignments included only conserved domains. The A and B subunits were analyzed separately and then concatenated for a partitioned analysis. Phylogenetic inference was performed using BEAST 2.7.6 (32). BEAST 2 analyses were conducted with the goal of estimating rooted trees, noting that without time calibrations, the analysis produces ages in units of substitutions per site, rather than absolute dates. The analyses used an (uncalibrated) optimized relaxed clock (from the ORC 1.2.0 package [33], the Yule skyline tree prior, BICEPS 1.1.2 [34], and the OBAMA substitution model, OBAMA 1.1.1 [35]). The Yule skyline model assumes that diversification rates vary through time in a smooth piecewise fashion, providing a model-based estimate. The conditional clade distribution 0 (CCD0) method was used to summarize the posterior distribution of trees (36). Tree entropy was measured to estimate phylogenetic information content in each posterior distribution of trees (18). Tree sets were visualized using DensiTree (37, 38). Phylogenetic trees were visualized using Figtree (http://tree.bio.ed.ac.uk/software/figtree/).

### Species phylogeny

We identified 16S rRNA gene sequences in the bacterial genomes using barrnap v0.9 (https://github.com/tseemann/barrnap/). This search identified genes from 197 of the 205 bacterial genomes. We then aligned these genes using MAFFT v7.4 (30, 31) and estimated the phylogeny using BEAST 2.7.6 (32). The latter was done using the bModelTest site and substitution model (39), with four gamma site rate categories (estimated posterior probability: 1.0) and invariant sites (probability: 1.0). We used the same clock model (33) (ORC 1.2.0) and tree prior (35) (Yule Skyline) as we did for the protein phylogenies and presented the CCD0 summary tree in this analysis (36).

### Ancestral sequence reconstruction

ModelFinder (40) from IQ-Tree (41) was implemented to identify the best evolutionary model for the MotA sequence alignment (LG + F + R10). Ancestral sequence inference for the MotA phylogenetic tree was performed using PAML 4.9 (42) (LG + F model). Sequence gaps were reconstructed by calculating the probability of a gap for each position based on a presence-absence sequence alignment and treating all positions with a gap probability ≥0.5 as a gap (43). Branch lengths were optimized based on the amino acid substitution models during ASR, to represent the expected number of amino acid substitutions per amino acid (42) (Fig. S5).

### Sequence conservation analysis

Flagellar and non-flagellar subunits were distinguished based on the clade distribution in the concatenated phylogenetic tree and reference to experimentally characterized systems (PDB IDs 6YKM, 8GQY, 5SV0, 8ODT). Sequence alignments for the flagellar and non-flagellar MotA homologs were constructed using MAFFT with iteration refinement over 1000 cycles. Residues falling within a defined conservation range, as determined by sequence alignment, were allocated transparency levels using UCSF-Chimera (44).

### *E. coli* strains, plasmids, and culture media for swim assays

Stator variants were tested in stator-deleted derivative strains of *E. coli* RP437 (*E. coli* ΔmotAB) (45). The pDB108 (pBAD33 backbone, Cm+) plasmid encoding *motA* and *motB* (46) was used as the vector to clone and express all constructs. Deletions in the plasmid-encoded *motA* gene were generated using inverse-deletion PCR. Primers are listed in Table S1. PCR was used to linearize the pDB108 plasmid, excluding nucleotides coding for the regions to be deleted. Linear PCR products were then re-ligated using T4 ligase (M0202S, New England Biolabs) and T4 polynucleotide kinase (Genesearch Pty Ltd) in T4 ligase buffer for 2 h at 37°C, followed by overnight incubation at 16°C. Every ligation reaction was then transformed into NEB10β competent cells (New England Biolabs). Plasmids were extracted from successful transformants for verification. Cloned constructs were confirmed by Sanger sequencing (Ramaciotti Center for Genomics, University of New South Wales, Kensington, Sydney, Australia). Liquid cell culturing was done using LB broth (NaCl or KCl, 0.5% yeast extract [70161, Sigma-Aldrich], and 1% Bacto tryptone). Cells were cultured in agar plates composed of LB broth and 1% Bacto agar (BD Biosciences, USA). Swim plate cultures were grown in the same substrates adjusted for agar content (0.25% Bacto agar) and NaCl (85 mM). Protein expression was induced from plasmids with 1 mM arabinose (Sigma, USA).

### Structural modeling

AlphaFold predictions were generated for all the proteins included in the phylogenetic analyses and for the ancestral proteins estimated with ASR. Models of stator monomers and heptameric complexes were produced with AlphaFold2 (47) supported by the Australian AlphaFold Service (https://www.biocommons.org.au/alphafold) on the public server at UseGalaxy.org.au (48). The software was run in multimer mode using FASTA files as input (47, 49). Models were visualized using PyMOL version 2.5.4 (50).

### Quantification of structural diversity across A-subunit homologs

Alphafold2 (AF2) monomeric models of all entries in the A-subunit phylogeny were manually inspected and classified as four-letter codes according to the following principles: the first letter indicates the presence (L for large) or absence (S for small) of amino acid residues at the N terminus of the TM domain; the second letter indicates the TGI domain was composed of three or more α-helices (L) or two or fewer (S). The third letter indicates whether the TM3 helix was broken in two (B) or unbroken (N). The fourth and last letter indicated the presence of one (S) or more (L) α-helices at the C-terminus of the protein.

### Characterization of plug and linker domains in MotB homologs

The length of the B subunit domain, defined as plug + linker (P + L), was estimated from the alignment of the 379 MotB homologs (277 non-flagellar and 102 flagellar entries). The Plug and Linker domains were defined as the residues between the catalytic TM domain and the peptidoglycan (OmpA-like) binding domain, based on the *E. coli* MotB (AAC74959.1) reference sequence SSPKELIQIAEYFRTPLATAVTGGDRISNSESPIPGGGDDYTQSQGEVNKQP after inspection of the MotA5B2 heptameric model generated using AF2. The sequence above corresponds to positions 76–133 of the alignment. The length and residue content of amino acid sequences in this portion of the alignment were calculated using MS Excel and plotted using GraphPad Prism.

## SUPPLEMENTAL MATERIAL

Supplemental Material mbio.03824-24-s0001.pdf Table S1, Figures S1 to S5, and caption for Table S2.

Table S2 mbio.03824-24-s0002.xls List of species and genome accession IDs.

ASM does not own the copyrights to Supplemental Material that may be linked to, or accessed through, an article. The authors have granted ASM a non-exclusive, world-wide license to publish the Supplemental Material files. Please contact the corresponding author directly for reuse.
