# Supplementary material for: Evolution and structural diversity of the MotAB stator: insights into the origins of bacterial flagellar motility
Source: mBio. 2025 Sep 10;16(10):e03824-24. doi: 10.1128/mbio.03824-24 (PMC12505986; doi:10.1128/mbio.03824-24)
Supplement: Supplemental Material — Table S1, Figures S1 to S5, and caption for Table S2. [file mbio.03824-24-s0001.pdf]

This document contains Supplemental Tables and Figures for the manuscript 'Evolution and Structural Diversity of the MotAB Stator: Insights into the origins of Bacterial Flagellar Motility', Puente-Lelievre et al.

Supplemental Data files are also available at:
https://github.com/carolinePL/MotAB-phylogeny.
Online files include PDBs for structural prediction as well as phylogenies for MotA, MotB, and MotAB in NEXUS format.

Primer Name	*Sequence (5’[illegible]3’)*
MotA dTGI5 Fw-124	*TTTATCGTCGATTATCTGCG*
MotA dTGI5 Rv-104	*ATTTTCAATATCACGTTCCAGC*
MotA dTGI5 Rv-105	*GGGATTTTCAATATCACGTTCC*
MotA dTGI5 Rv-108	*GCTCTCACGGGGATT*

Supplementary Table 1. Primers used for cloning truncated variants of *motA* in plasmid pDB108. Note that truncated construct MotA Δ6 (Δ103-124) was accidentally isolated while screening for successfully cloned constructs and was added to the panel of MotA truncations to test for motility.

Supplementary Table 2. List of genomes, accession IDs and taxonomic information for all genomes used in this work.

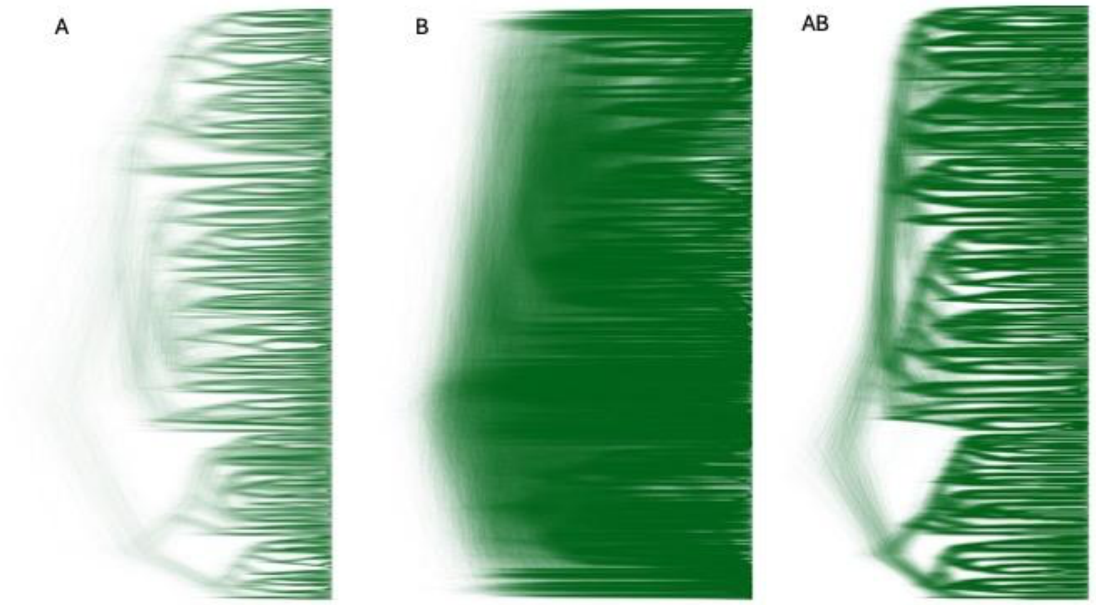

Figure S1. DensiTree visualization of posterior tree distributions inferred from BEAST2 analyses. A) posterior tree distribution for A subunits; B) posterior tree distribution for B subunits; AB) posterior tree distribution for subunits AB concatenated. Each panel shows a density plot of the sampled posterior trees, where line density and sharpness indicate the level of topological certainty. Well-supported clades appear as sharp, dark branches, while regions of topological uncertainty appear as diffuse, fuzzy areas. The comparison highlights differences in clade support and topological resolution between individual datasets and the combined analysis.

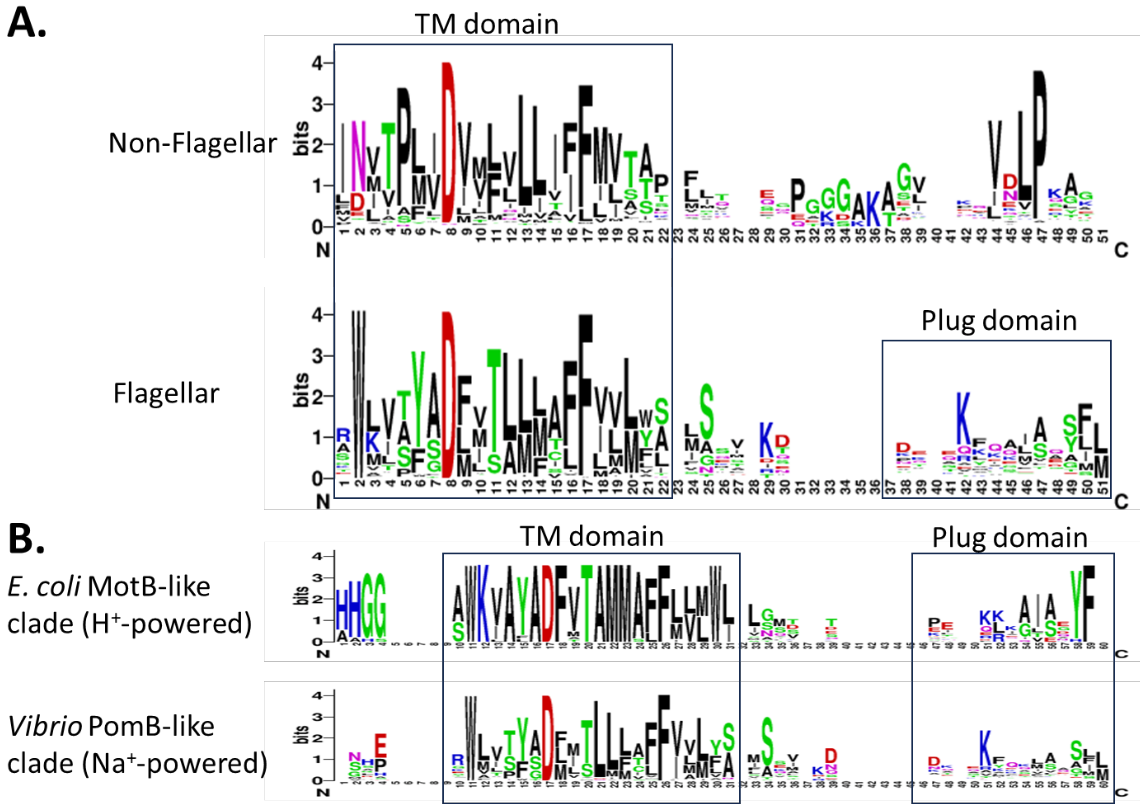

Figure S2. Residue conservation comparison between the FIT and GIT B subunits. A) Consensus logos are shown for the TM domain and Plug domain portions of B-subunit homologs belonging to Non-Flagellar (top) or Flagellar (bottom) clades. B) Consensus logos of the TM and Plug domains regions of homologs belonging to the same clade as the proton-powered *E. coli* MotB (top) or sodium-powered *Vibrio* PomB (bottom).

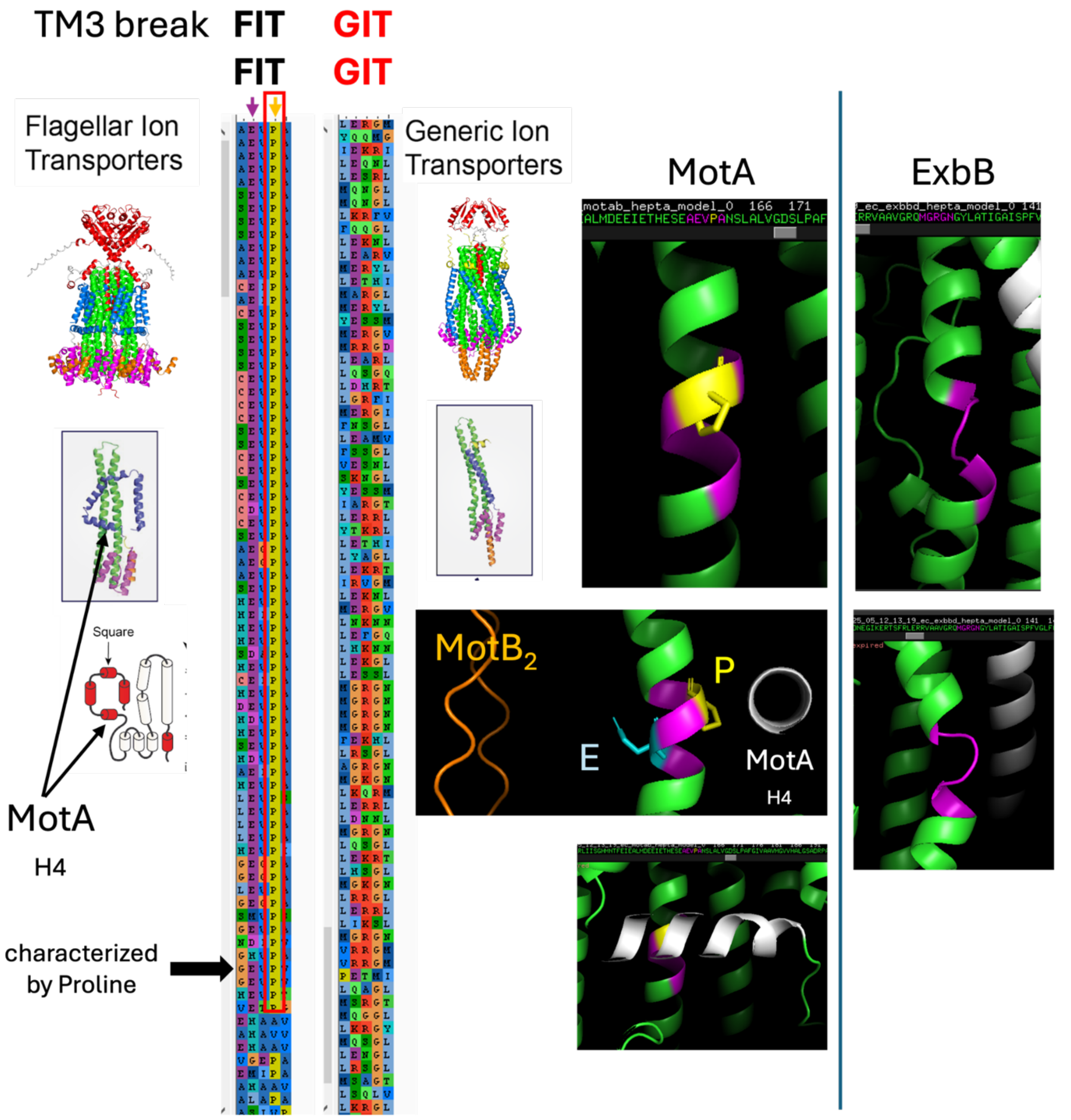

Figure S3. Comparative analysis of TM3 structural divergence between Flagellar Ion Transporters (FIT) and Generic Ion Transporters (GIT). Local alignments of residues surrounding the transmembrane helix 3 (TM3) reveal conserved differences between FIT (left) and GIT (right) proteins. In FIT proteins, a highly conserved Glu–Pro (E–P) motif (highlighted residues) stabilizes TM3, positioning glutamic acid (E, cyan) toward the MotB subunit (orange) and proline (P, yellow) toward the adjacent helix (H4) of MotA. This arrangement likely contributes to structural rigidity critical for torque generation. In contrast, GIT proteins exhibit a TM3 break at proline residues, flanked by positively charged arginine (R) and lysine (K) residues (magenta), resulting in a kinked helix exposed to the solvent and lacking stabilizing interactions. Structural snapshots (right) illustrate these differences, highlighting distinct evolutionary trajectories linking ion passage to specific conformational changes and biological functions in FIT versus GIT lineages.

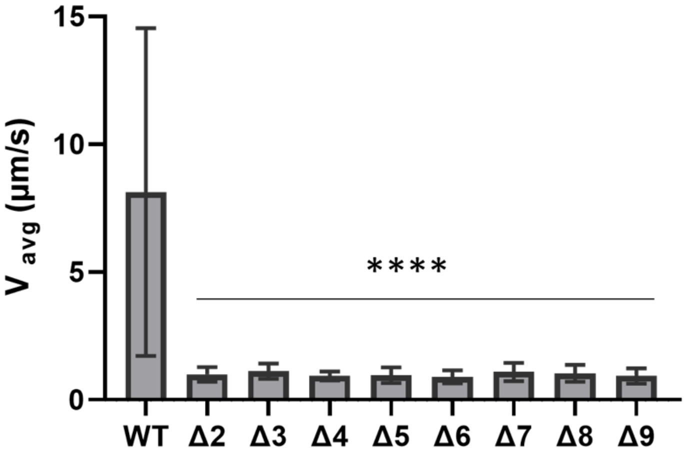

Figure S4. Average speed of free-swimming single cells in LB broth supplied with 1mM Arabinose, for wild-type and eight variants of *Ec*MotA with partially deleted TGI5 domains. Deletions spanned residues N103 – D124; WT MotA (N = 230 cells); Δ2: 105-123 (N = 333 cells); Δ3:107-123 (N = 267 cells); Δ4:108-123 (N = 148 cells); Δ5:104-124 (N = 295 cells); Δ6:103-124 (N = 324 cells); Δ7:105-124 (N = 320 cells); Δ8:107-124 (N = 318 cells); Δ9:108-124 (N = 353 cells). Error Bars indicate standard error of the mean. Statistical analysis was performed using pairwise Student's T test; **** P value < 0.0001.

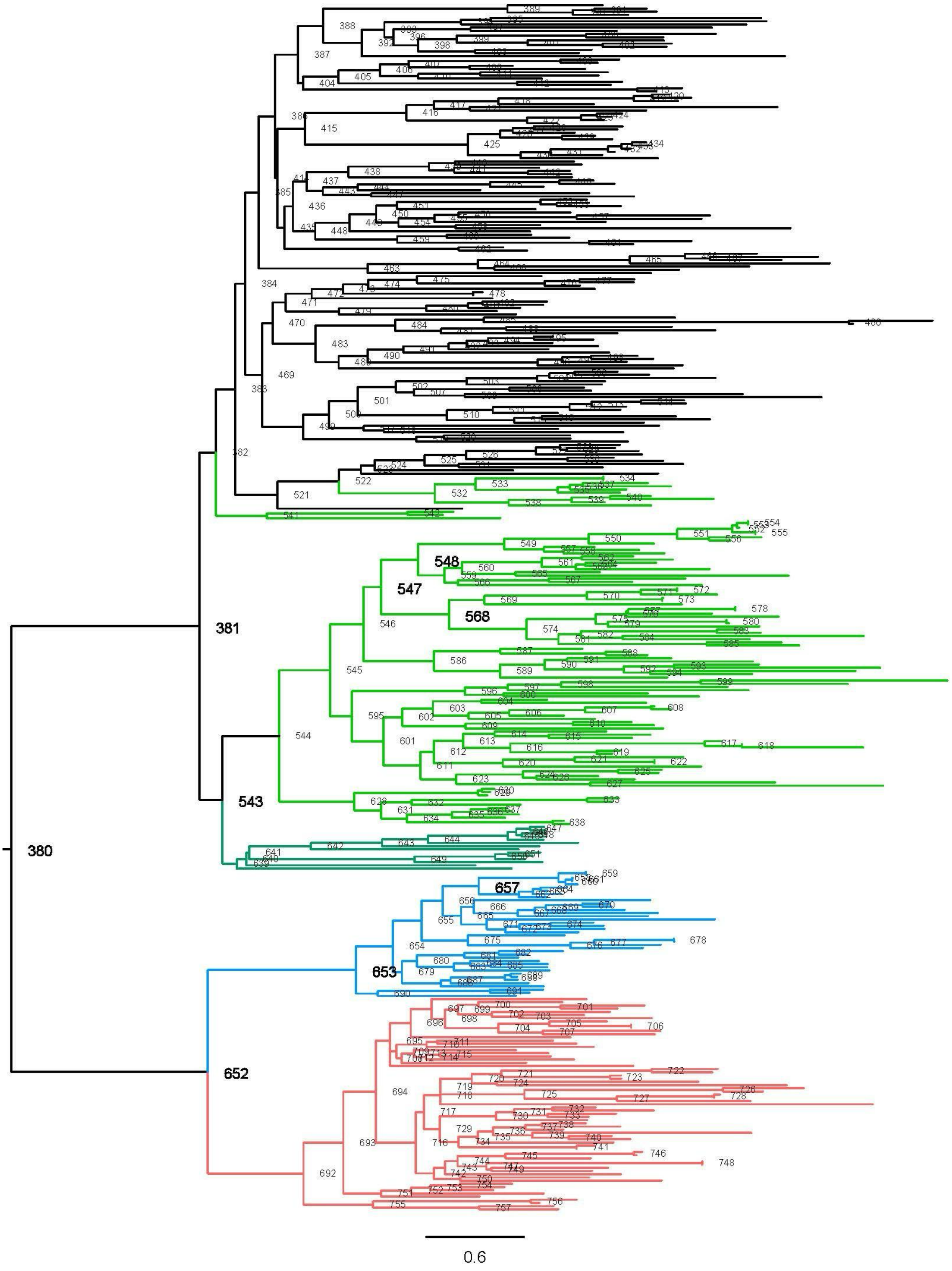

Figure S5. Ancestral sequence reconstruction phylogeny of the A subunits colored based on main structural groups. TGI4: red, TGI5: blue, CCD2*: dark green, CCD2: lighter green, CCD3: black. The nodes with structure predictions represented in Figure 7 are boldened in larger font.

Supplementary MSA 1. Multiple sequence alignment (MSA) for MotA used to calculate Supplementary Phylogeny 1 (MotA).

Supplementary MSA 2. Multiple sequence alignment (MSA) for MotB used to calculate Supplementary Phylogeny 2 (MotB).

Supplementary MSA 3. Multiple sequence alignment (MSA) for concatenated MotAB used to calculate Supplementary Phylogeny 3 (MotAB).
